# A new species of sponge inhabiting barnacle *Bryozobia* (Archaeobalanidae, Bryozobiinae) in the West Pacific

**DOI:** 10.3897/zookeys.571.6894

**Published:** 2016-03-07

**Authors:** Meng-Chen Yu, Gregory A. Kolbasov, Benny K.K. Chan

**Affiliations:** 1 Biodiversity Research Center, Academia Sinica, Taipei 11529, Taiwan; 2 Doctoral Degree Program in Marine Biotechnology, National Sun Yat-sen University and Academia Sinica, Kaohsiung 80424, Taiwan; 3 White Sea Biological Station, Biological Faculty, Moscow State University, 119991, Moscow, Russia

**Keywords:** Sponge inhabiting barnacle, Archaeobalanidae, Bryozobiinae

## Abstract

This paper describes a new species, *Bryozobia rossi* **sp. n.**, collected by scuba diving in both Taiwan and Japan. *Bryozobia rossi* **sp. n.**, a member of the subfamily Bryozobiinae (Ross and Newman 1996), has atria and open end portals and a single irregular basal whorl of portals at the same level as basal hemiportals; this morphology varies from all previously described bryozobiines. According to our review of relevant literature, this is the first reported *Bryozobia* in the Pacific, and this study is the first to describe the morphology of oral cone, cirri, and penis for the genus *Bryozobia*.

## Introduction

**Figure 1.**
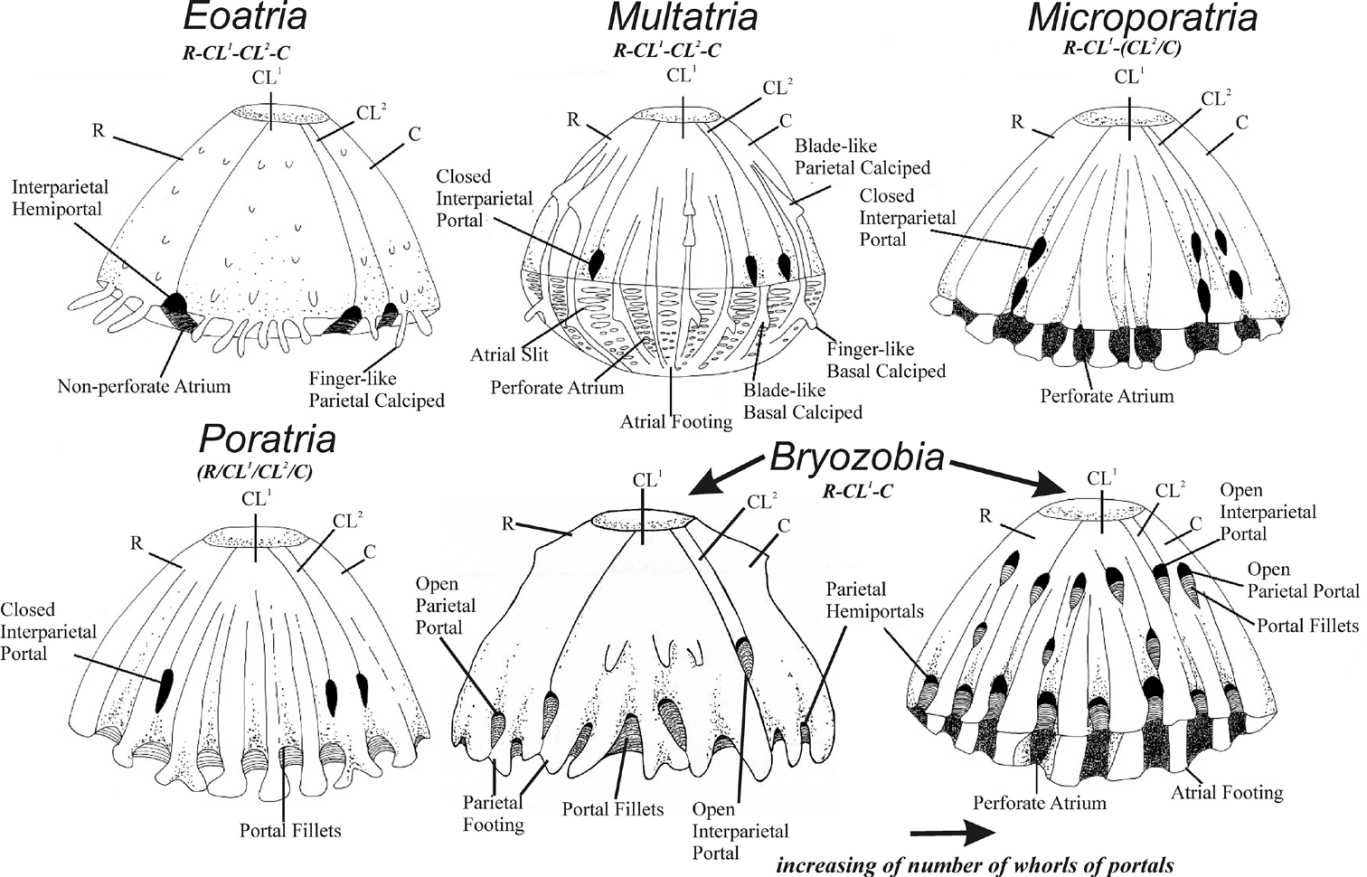
Diagrammatic representations of 5 genera of Bryozobiinae, with indication of plate formula for basal portion of shell in adults (see explanations in text). Modified from Van Syoc and Newman 2010.

Barnacles of the subfamily Bryozobiinae are considered obligate symbionts of sponges attaching to various calcareous substrates, such as mollusk shells, bryozoans, corals. Morphologically, bryozobiines are unique in remaining attached to sponges substrates and possessing calcareous portals and atria (openings and tubular arched passages) in their base and walls (Table 1 and Figure 1; Van Syoc and Newman 2010). The number of plates in the shell base, determined by either the partial or complete fusion of plate sutures in the shell base or the elimination of short carinolaterals^2^ (CL^2^) that do not reach the base, varies from six to four. These barnacles can modify the external shell structure with atria and portals, thus creating additional chambers that allow the growth of encrusting or burrowing sponges (Gregg 1948; Pilsbry 1916; Ross and Newman 1996; Van Syoc and Newman 2010).

**Table 1.** Glossary of nomenclature relevant to Bryozobiinae. Modified from Van Syoc and Newman 2010.

Terms	Explanation	Types	Explanations
Atria	Arched chambers or passages of calcareous basis, radiating from center and opening to exterior with hemiportals and portals.	Atrial Footing	Footing area of basis
		Non-perforate Atria	Solid atria without pores
		Perforate Atria	Atria perforated with small pores
		Slit Atria	Atria perforated with elongated slits
Calcipeds	Calcareous projections of shell exterior of different shape.	Finger-like Parietal Calcipeds	Finger-shaped projections of parietal wall
		Finger-like Basal Calcipeds	Finger-shaped projections of basis
		Blade-like Parietal Calcipeds	Blade-shaped projections of parietal wall
		Blade-like Basal Calcipeds	Blade-shaped projections of basis
Portal	Openings on shell connected or not connected or not with atria, may be arranged in several whorls and elevated with growth of shell or not.	Portal Fillets	Sliced passage opening
		Interparietal (Sutural) Hemiportal	Non-encircled passage opening at basal part of wall plates suture
		Interparietal (Sutural) Portal	Encircled passage opening between wall plates at sutural area.
		Parietal Hemiportal	Non-encircled passage opening at base of parietes
		Parietal Portal	Encircled passage opening removed from base of parietes
			Portals connected with basal atria via arched fillets
		Open portals	Portals lost connection with basal atria
		Closed portals	
Footing	Massive processes of basis or basal part of parietes	Parietal Footing	Massive basal processes of parietes
		Atrial Footing	Massive processes of basis between atria

The subfamily Bryozobiinae and the type genus *Bryozobia* were first described by Ross and Newman (1996) using samples collected from Madagascar and Mauritius; they reported *Bryozobia* as an obligate symbiont of bryozoans. The unique characteristics of the subfamily Bryozobiinae are the unusual perforate calcareous tubes and passages (atria) in the shell wall and base (Table 1), in which the bryozoan tissue extends through the cavity. The genus *Bryozobia* is characterized by atria that open with portals arranged in two or three whorls resulting in a four-plated wall by eliminating CL^2^ at the shell base. Subsequently, Van Syoc and Newman (2010) re-established the subfamily Bryozobiinae to include four additional genera, *Eoatria*, *Microporatria*, *Multatria*, and *Poratria*, which are obligated symbionts of sponges instead of bryozoans and additionally attach to various calcareous substrates including mollusks and corals. Van Syoc et al. (2015) revealed that bryozobiine species are commonly obligated on encrusting sponges.

Currently, Bryozobiinae consists of five genera and ten species. The shell structure of all species possesses calcareous tubular passages or atria of the base remaining attached to the substratum. The number of shell plates and their fusion/elimination at the base and the structure of atria and portals (Table 1, Figure 1) are diagnostic morphological characters of bryozobiines. Genus *Eoatria* possesses six interparietal hemiportals, six separate shell plates of similar length, and a nonperforate base. *Multatria* has six separate shell plates of similar length with a whorl of six interparietal basal portals between them and a perforate base. All six shell plates of *Poratria* are fused at the base, with a primary whorl of six interparietal portals and numerous basal portals and hemiportals, and a perforated base. *Microporatria* has CL^2^ fused with the carina at the base of sutures below the portals; therefore, the shell has four plates in the base and a perforated base. Genus *Bryozobia* is characterized by smaller CL^2^ eliminated with interparietal portal of the first elevated whorl; therefore, the shell has four plates in the base, and the portals form several whorls and remain attached to the basal atria through arched fillets (open portals). However, other bryozobiine portals dissociate from the basal atria during growth and elevation.

Only a single species *Bryozobia synaptos* (Ross and Newman 1996) from Madagascar and Mauritius was described for the genus *Bryozobia* (Ross and Newman 1996, Van Syoc et al. 2015). Recently, a few undetermined juveniles of *Bryozobia* sp. were found on a gastropod shell in Sri Lanka without description of opercula and a soft body (Van Syoc and Newman 2010). The soft tissue of *Bryozobia* was unknown as only available material was sub-fossil; therefore, the descriptions were incomplete.

In the present study, we collected several living bryozobiines from Green Island and Orchid Island (Taiwan) and Kochi (Japan) with only an irregular whorl of shell portals and remained attached to the basal atria through arched fillets and smaller CL^2^ eliminated by interparietal portal. These characters suggest that this is a new species of genus *Bryozobia* and the presence of soft bodies completes the description of this genus.

## Material and methods

**Figure 2.**
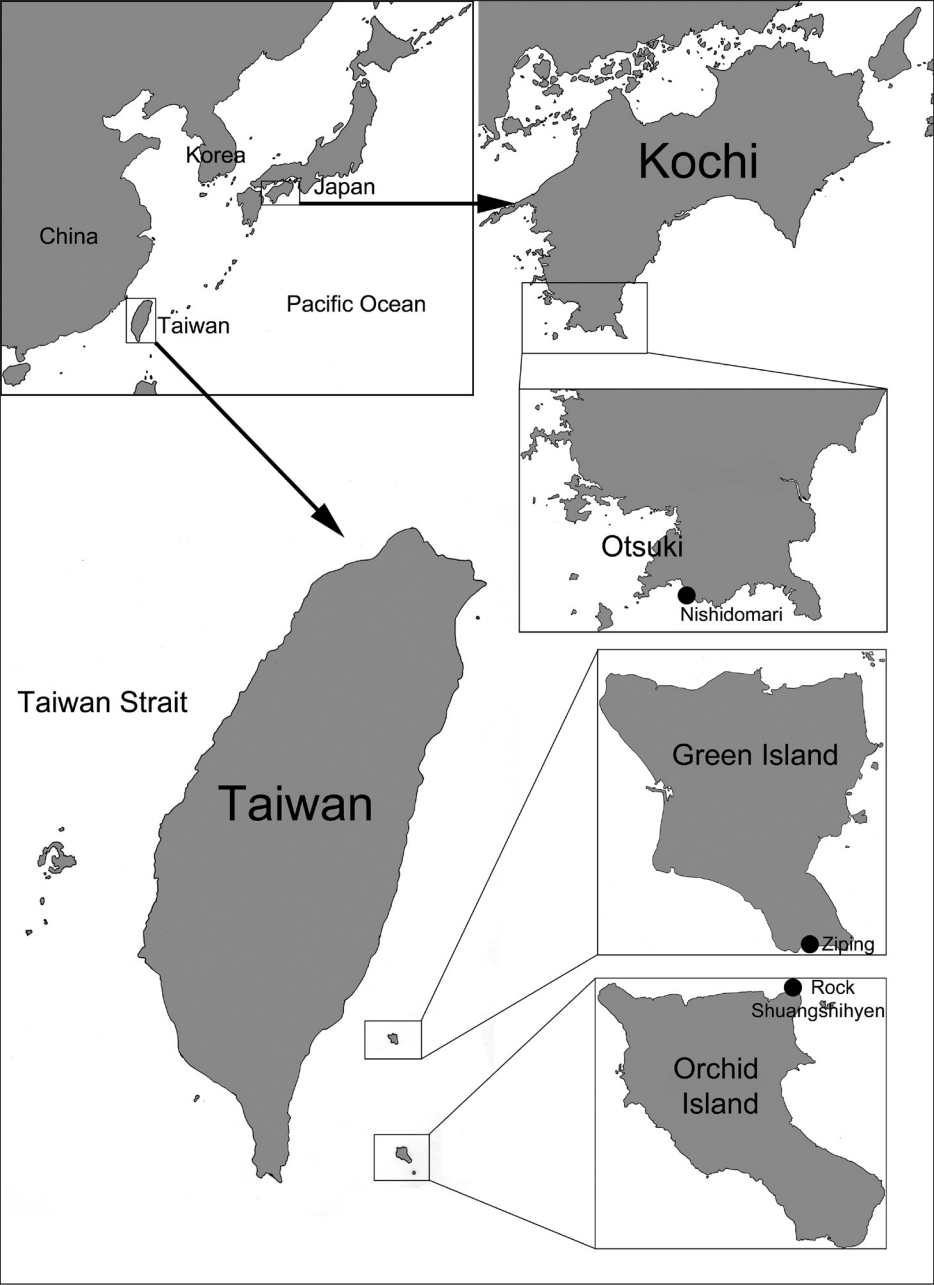
Collection sites of sponge-inhabiting bryozobiine barnacles in Taiwan and Japan.

Bryozobiines were collected from thin encrusting sponges on rocks (*Agelas nakamurai* Hoshino 1985, Theonella aff. conica Kieschnick 1896, and *Theonella mirabilis* [de Laubenfels 1954]) in Taiwan (Green Island and Orchid Island) and Japan (Kochi) by scuba diving to a depth of 3–24 m (Figure 2). Barnacles were separated from the host sponges using forceps and 95% EtOH was injected into mantle cavity for better fixation of the soft tissue for molecular analysis, in prior to the whole specimen was immersed in Ethanol. Both the barnacle and sponges were subsequently preserved in 95% EtOH. Morphological characters of barnacle shell parts (basis, plates, scutum, and tergum) and somatic bodies (six pairs of cirri, the penis, and oral cone) were examined. The remnants of the sponge on the surface of shell, scutum, and tergum were removed using forceps and immersed in 2% bleach for about two hours to completely digest the organic tissue and rinsed subsequently in purified water for five times and air-dried. The shell, scutum, and tergum were observed under stereomicroscope Leica MZ 6 (Leica, Germany) and digital single-lens reflex cameras (Canon EOS 5D Mark III, Canon Camera Co. Ltd, Japan) installed with a 65 mm f/2.8 1–5× macro lens. Then shell, scutum and tergum were air-dried, gold-coated and observed under SEM, following methods in Chan et al. (2013).

All six pairs of cirri, penis, and oral cone were dissected from the somatic bodies, and the organic debris were removed using forceps and an ultrasonic cleaner (for 1–3 seconds) and examined through light microscopy (Zeiss Scope A1, Zeiss, Germany) using high-definition lenses (Zeiss Plan APO Chromat 40X/0.95) to clearly observe the setae types on the cirri and the mouthparts.

The glossary of nomenclature relevant to Bryozobiinae and setae morphology were described according to Van Syoc and Newman (2010) and Chan et al. (2008), respectively. The holotype and the paratypes were preserved at the Biodiversity Research Museum of Academia Sinica, Taipei, Taiwan (ASIZCR) and the Zoological Museum of Moscow State University (Mg) whereas the additional specimens were preserved at the barnacle collection of the Coastal Ecology Lab (CEL), Academia Sinica, Taiwan. The specimens of sponge were preserved at the National Penghu University of Science and Technology Porifera Collection (NPUST; POR).

## Systematics

### Suborder Balanomorpha Pilsbry, 1916 Superfamily Balanoidea Leach, 1817 Family Archaeobalanidae Newman & Ross, 1976 Subfamily Bryozobiinae Ross & Newman, 1996 Genus *Bryozobia* Ross & Newman, 1996

#### Bryozobia rossi

Taxon classificationAnimaliaSessiliaArchaeobalanidae

Yu, Kolbasov & Chan sp. n.

http://zoobank.org/3BD4CEC0-0D6B-4F0B-A1DA-E37D287F0ADA

Figures 3 , 4 , 5 , 6 , 7 , 8 , 9 , 10 , 11 , 12

##### Type species.

*Bryozobia synaptos* Ross & Newman, 1996

##### Materials examined.

Holotype: Taiwan, Taitung, Green Island (Lyudao), Ziping, 22°37.99'N, 121°29.99'E, depth 24 m, November 15, 2011, coll. J.H.Y. Yu, ASIZCR-000338, on host sponge *Agelas nakamurai* (Hoshino, 1985), NPUST.POR.0357.

Paratypes: ASIZCR-000339, ASIZCR-000340 and Mg. 1222

##### Other materials.

Taiwan, Taitung, Orchid Island (Lanyu Island), Rock Shuangshihyen, 22°05.14'N, 121°34.10'E, depth 24 m, June 11, 2011, coll. J.H.Y. Yu, CEL-SOI33-1, on host sponge Theonella aff. conica (Kieschnick, 1896), NPUST.POR.0354.

Other materials: Japan, Nishidomari, Kochi, 32°46.48'N, 132°43.89'E, depth 5 m, July 22, 2011, coll. J.H.Y. Yu, CEL-SJP5-1, on host sponge *Theonella mirabilis* (de Laubenfels, 1954), NPUST.POR.0350.

##### Diagnosis.

Shell with unfused sutures, external surface with a few calcipeds and indistinct longitudinal ribs, vestige of CL^2^ with elevated interparietal portal on each side, an irregular whorl of open portals, and edges of parietal footings that may merge to completed portals. Calcareous base, base flat or saucer-shaped with numerous radial atria (app. 24) permeated by dense, irregularly shaped pores. Scutum with a prominent articular ridge, articular furrow low, concave pits of adductor and depressor muscles. Broad tergum with a beak-shaped apex, high and short articular ridge, and sloping spur.

##### Description.

**Figure 3.**
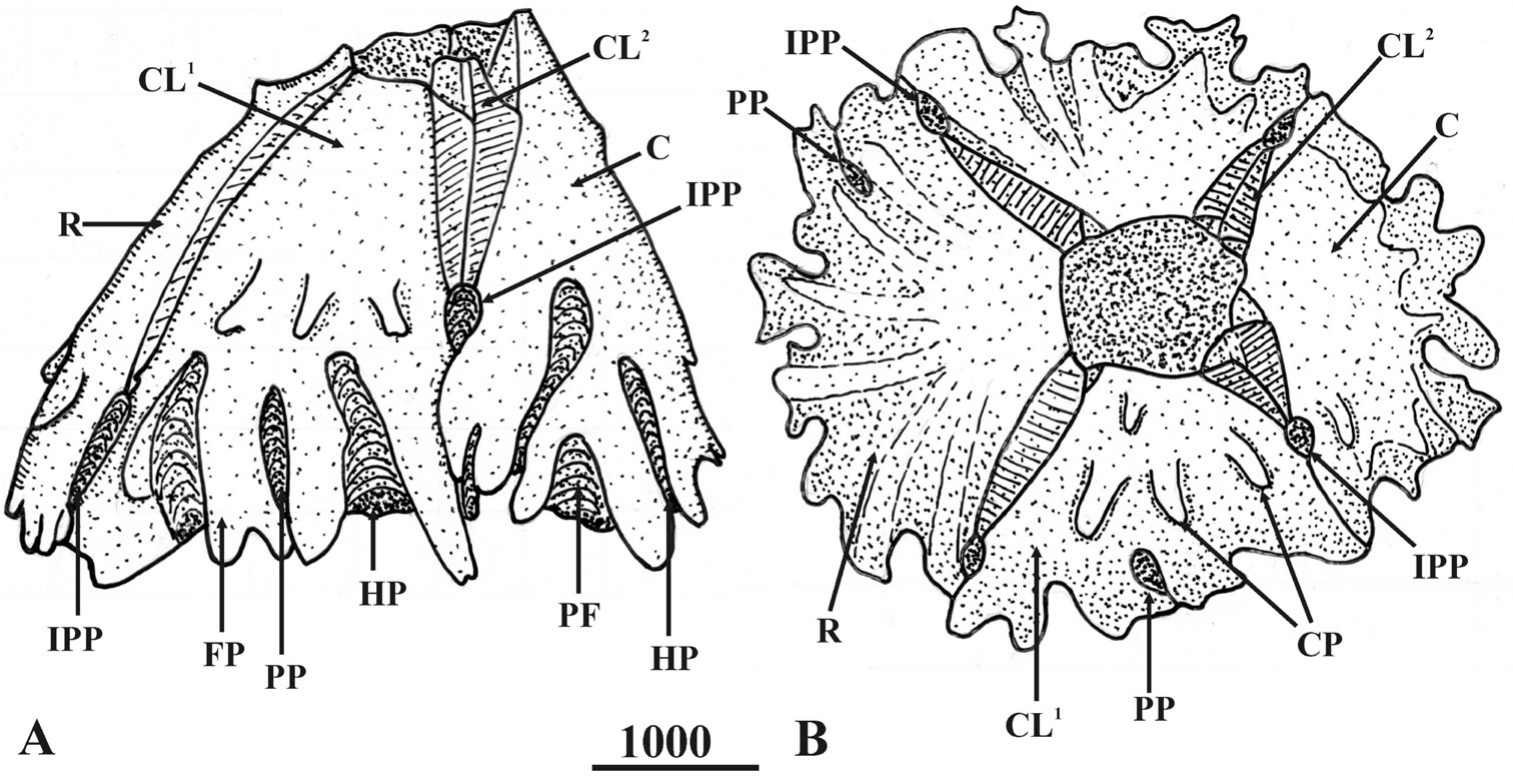
*Bryozobia rossi* sp. n., shell (opercular plates removed), general morphology. **A** general view, lateral side **B** top view. Abbreviations: C, carina; CL^1^, carinolateral^1^; CL^2^, carinolateral^2^; CP, calcipeds; FP, parietal footing; HP, hemiportals; IPP, interparietal portal; PF, portal fillets; PP, parietal portal; R, rostrum. Scale bar in µm.

**Figure 4.**
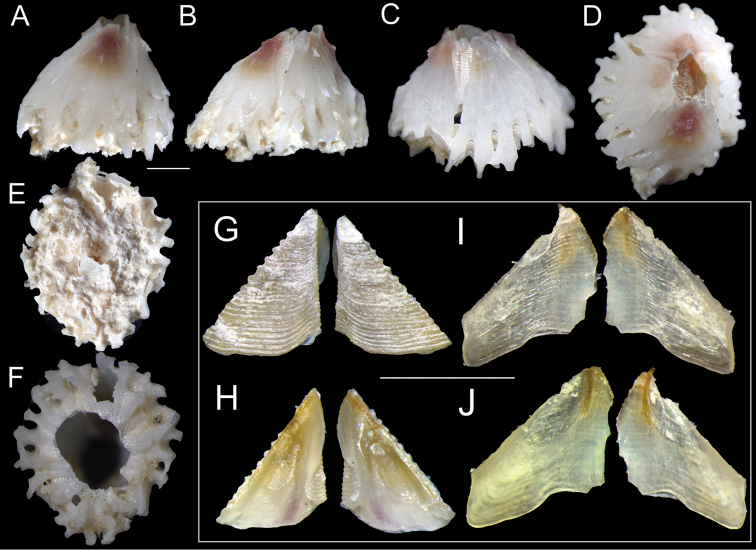
*Bryozobia rossi* sp. n. CEL-SJP5-1. Complete shell, scuta and terga. **A** rostral view **B** lateral view **C** carinal view **D** top view **E, F** basal view, sponge remnants and central part of basis removed in ‘**F**’ showing structure of basis **G** external view of scuta **H** internal view of scuta **I** external view of terga **J** internal view of terga. Scale bars: 1 mm.

**Figure 5.**
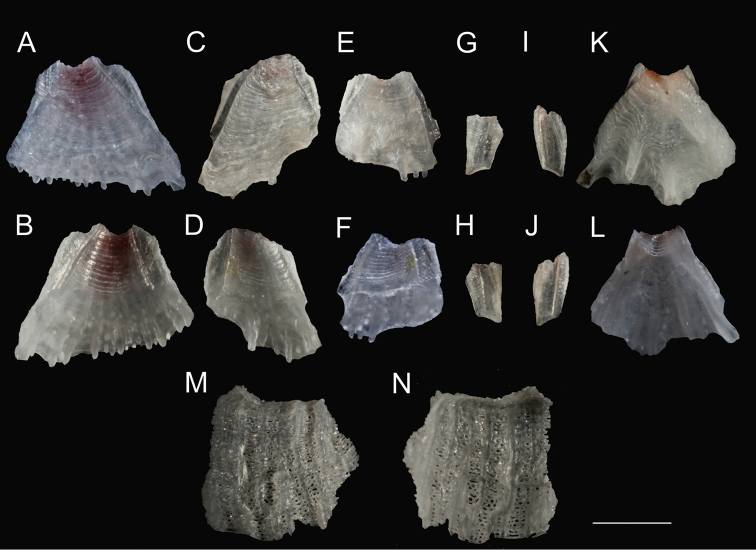
*Bryozobia rossi* sp. n. CEL-SOI33-1.Disassembled shell showing separated plates and part of basis after bleach treatment. **A, B** external and internal view of rostrum **C, E** external view of carinolaterals^1^ **D, F** internal view of carinolaterals^1^ **G, I** external view of carinolaterals^2^ **H, J** internal view of carinolaterals^2^ **K, L** external and internal view of carina **M, N** external and internal view of part of basis. Scale bar: 1 mm.

**Figure 6.**
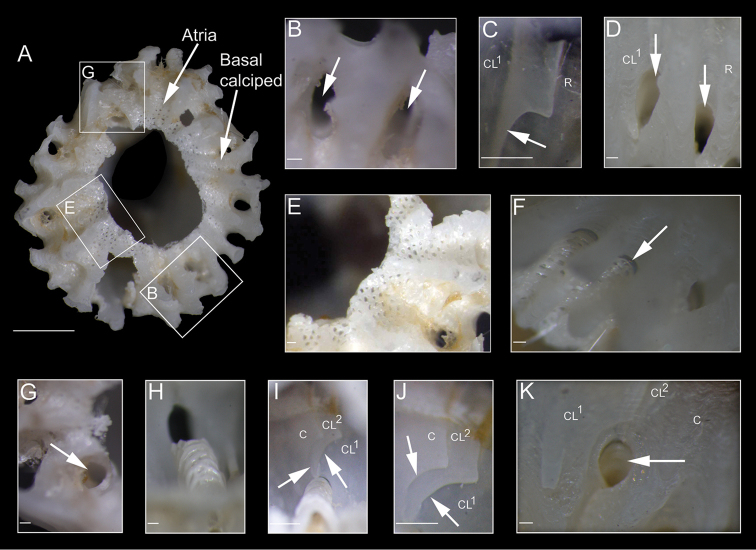
*Bryozobia rossi* sp. n. CEL-SJP5-1. **A** basal view of shell showing basis (central part destroyed) with atria and basal calcipeds **B** part of margin of basis with two atria and their portals (indicated by arrows) **C** suture (indicated by arrow) between CL^1^ and R, interior view **D** exterior view of part of shell with interparietal and parietal portals (indicated by arrows) in basal parts of CL^1^ and R **E** enlarged part of basis with porous atria **F** enlarged fillets of hemiportals (indicated by arrow) showing porous and sliced structure between basis and parietes, external view **G** basal view of interparietal portal opening **H** enlarged broken atrial fillet (tube) showing sliced structure (inner side of shell) **I** sutures between CL^1^, CL^2^ and C (indicated by arrows) and fillet of interparietal portal eliminated CL^2^, interior view **J** enlarged sutures (indicated by arrows) between CL^1^, CL^2^ and C **K** exterior view of interparietal portal eliminates CL^2^. Abbreviations: C, carina; CL^1^, carinilateral^1^; CL^2^, carinolateral^2^; R, rostrum. Scales: 1 mm (**A**); 0.1 mm (**B–K**).

**Figure 7.**
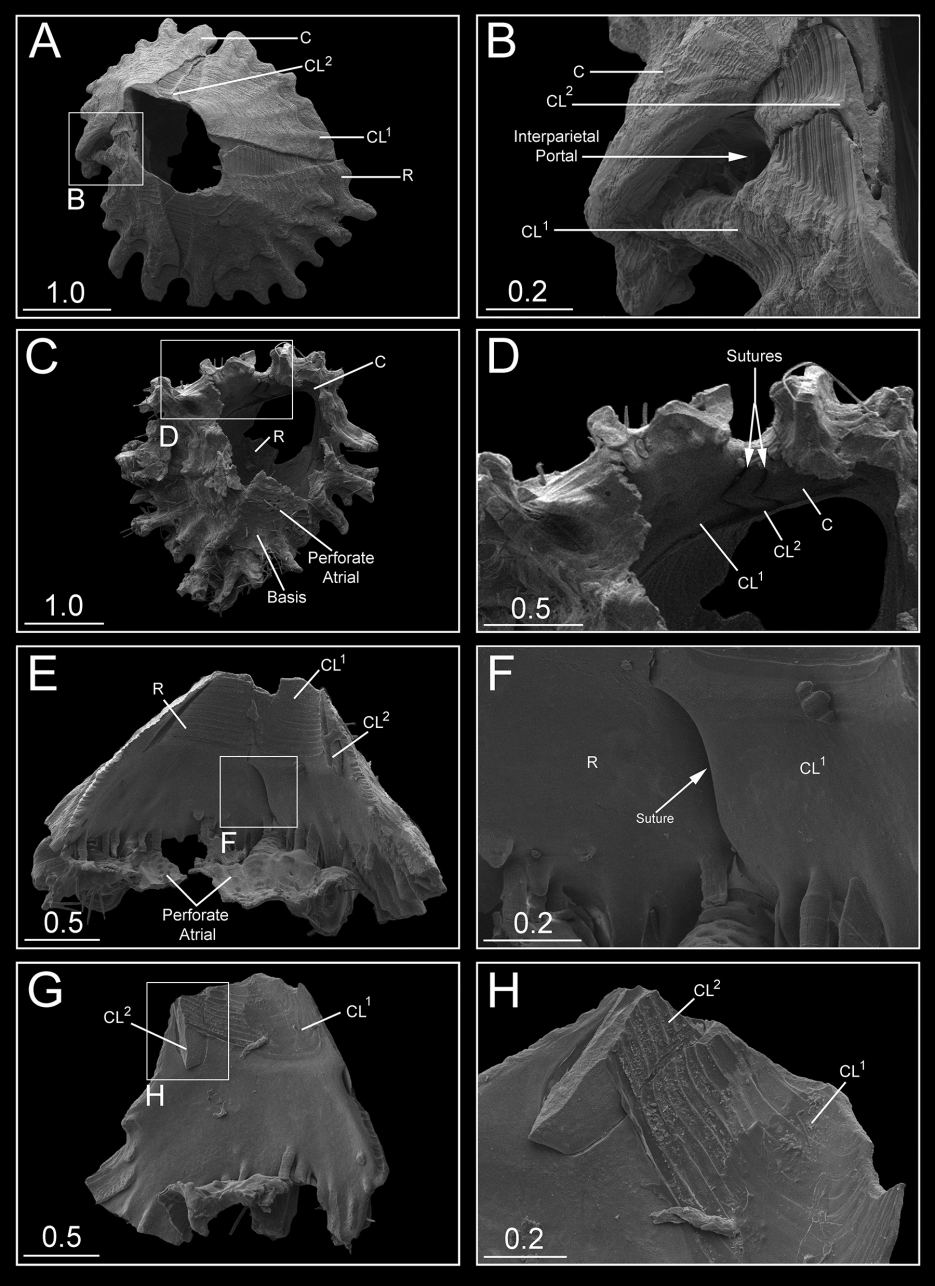
*Bryozobia rossi* sp. n. **A** ASIZCR-000338, Top view of shell showing unfused wall plates **B** ASIZCR-000338, enlarged external area of shell showing CL^2^ eliminated by interparietal portal **C** ASIZCR-000339, basal view of shell with partially destroyed basis **D** ASIZCR-000339, internal view of wall plates showing unfused sutures between CL^1^, CL^2^ and C **E** ASIZCR-000340, internal view of wall plates with basal longitudinal ribs and basis fragment with perforated atria **F** ASIZCR-000340, enlarged part of inner wall surface with unfused suture between R and CL^1^ **G** ASIZCR-000340, interior view of fragment of CL^1^ and CL^2^ **H** ASIZCR-000340, enlarged view of inner suture between CL^1^ and CL^2^. Abbreviations: C – carina, CL^1^ – carinilateral^1^, CL^2^ – carinolateral^2^, R – rostrum. Scale bars in mm.

White shell, tinged pinkish toward apex, with a maximal height range of 3–3.7 mm, basal diameter range of 3.3–4.6 mm, orifice range of 1.0–1.3 mm, and six plates (R-CL^1^-CL^2^-C) with unfused sutures, roughened and plicated exterior parietes with fine growth lines and few finger- and blade-like calcareous calcipeds on the surface (Figures 3, 4A–D, 5A–L, 6C, D, F, I–K, 7); smooth and digitate longitudinal ribs in the parietes base extending to the parietal footings that may merge and form completed portals (Figures 3, 4A–F, 6A, B, D, F, G, 7C–E); a whorl of rare interparietal and parietal portals in the shell base, two interparietal portals below rudimentary CL^2^ slightly elevated; plates eliminated at half the total length of the shell (Figures 3, 4C, D, 5G–J, 6B, D, G, H, I, K, 7B, D, G, H). All portals were open and attached to the basal atria through arched sliced fillets (Figures 3, 4C, D, F, 6A, B, D, G, H, I, K, 7B). All plates, except CL^2^, were wide and triangular, with irregular basal margins and internal longitudinal ribs rostrum being the biggest (Figures 3, 4A–D, 5A–L, 6 A, I, K, 7A–E, G, H). The smallest CL^2^ were irregularly rectangular two–three times shorter than other plates (Figures 3, 4C, D, 5G–J, 6I, J, K, 7A–E, G, H). Radii transparietal, summits slightly oblique, triangular, solid, horizontally striated (Figures 3, 4B–D, 5A–J, 7A, B). Alae developed in the summits almost horizontally. Sheath developed approximately one-fifth in the carina and one-third to one-half of the total height in other plates (Figures 5A–L, 7E–H). Calcareous base, flat or saucer-shaped with less than twenty atria, atria width approximately 0.1 mm, permeated by irregularly shaped small and dense pores were solid, radial, and indistinct calcipeds with separated atria, radiating from the center and extending out to basal margin and attached to the longitudinal ribs of parietes (Figures 4E, F, 5M, N, 6A, E, 7C, E).

Externally, scutum (Figure 4G, H) with horizontal growth ridges, without longitudinal striation; teeth present in the upper half of occludent margin; slightly bisinuous basal margin, strongly prominent articular ridge, approximately two-thirds the height of articular margin, articular furrow low, central adductor ridge, short, feeble, faint, and long depression for adductor muscle, and deep depressor and rostral muscles pits, lie directly at the basal margin. Tergum (Figure 4I, J) thin and semitransparent, nearly flat, with a beak-shaped apex; short and prominent articular ridge, broad articular furrow, without crests of depressor muscles; sloping spur not distinctly separated from the basiscutal angle of scutal margin, width approximately half of the basal margin, acute basiscutal angle; basal margin concave in the middle, wide and shallow spur furrow.

**Figure 8.**
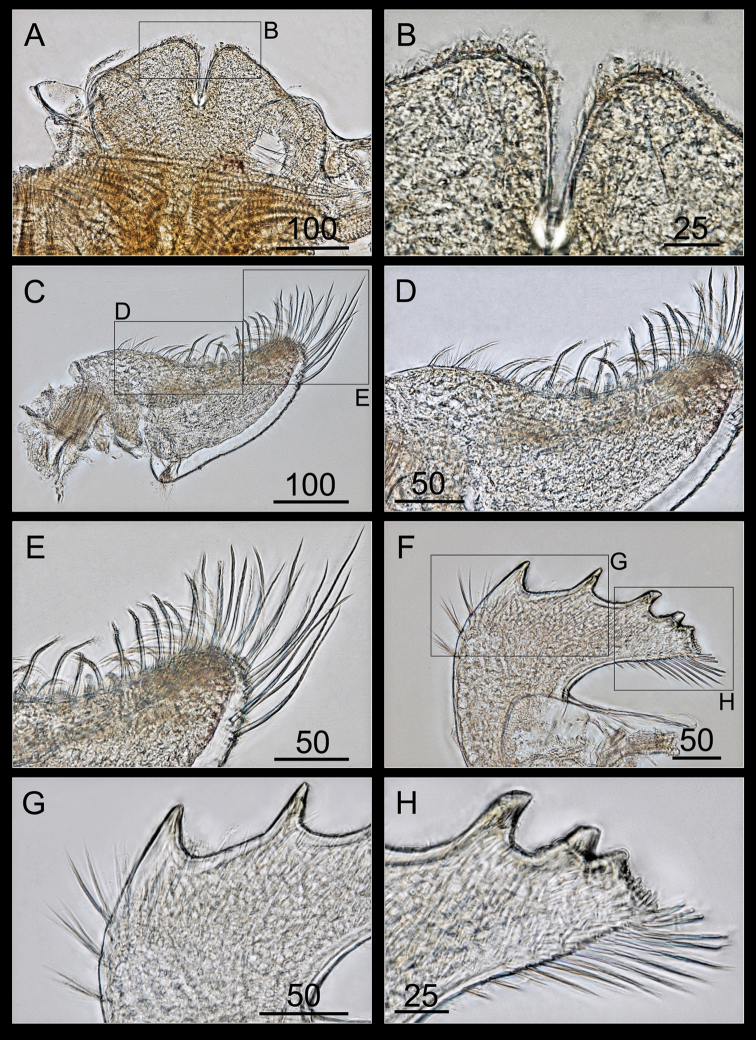
*Bryozobia rossi* sp. n. ASIZCR-000338. Labrum (**A, B**), mandibular palp (**C–E**) and Mandible (**F–G**). **A** labrum **B** teeth on crests **C** mandibular palp **D** outer margin **E** serrulate setae on distal part **F** mandible **G** upper part with bifid second teeth **H** inferior angle. Scale bars in μm.

Labrum bilobed, separated by deep V-shaped notch (Figure 8A, B), with two or three small teeth on each side of the crest (Figure 8B).

Mandibular palp ovate with concave outer margin (Figure 8C, D), dense serrulate setae along the outer margin and tip (Figure 8E).

Mandible with five teeth (Figure 8F), second and third teeth bifid, upper margin bearing simple setae (Figure 8G) and the inferior angle ending in blunt angle with stout simple setae (Figure 8H).

**Figure 9.**
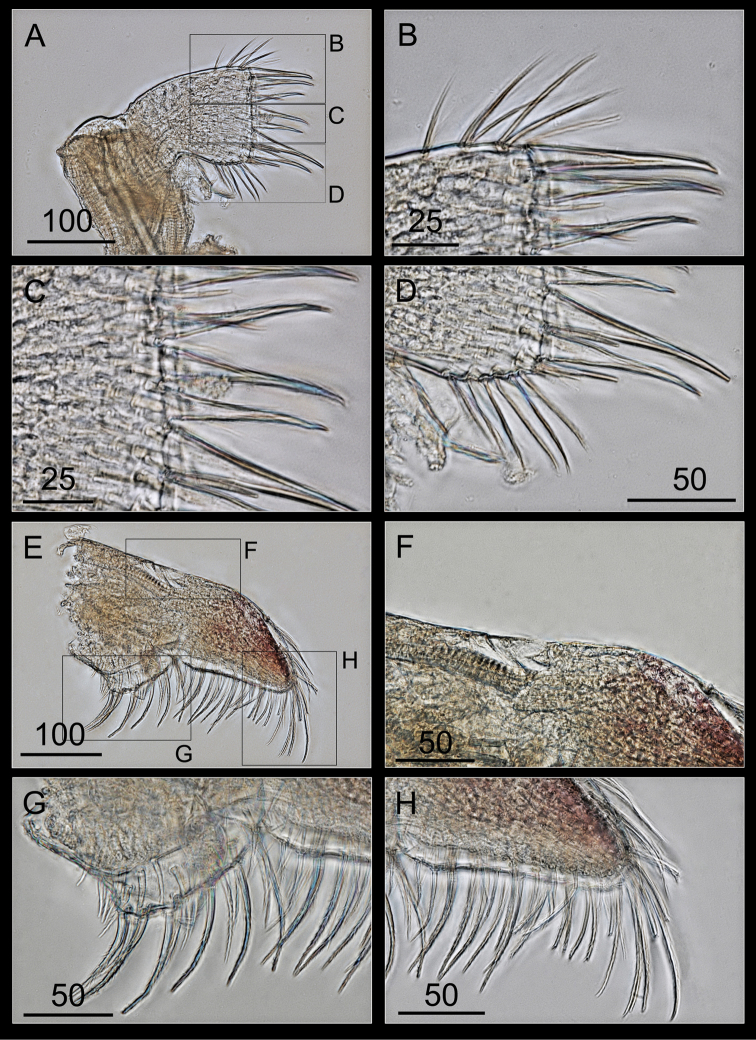
*Bryozobia rossi* sp. n. ASIZCR-000338. Maxillule (**A–D**) and maxilla (**E–H**). **A** maxillule **B** upper part of cutting edge **C** straight cutting edge **D** lower part of cutting edge **E** maxilla **F** outer edge of distal lobe **G** inner edge of distal lobe **H** terminal part of distal lobe. Scale bars in μm.

Maxillule with a straight cutting edge and seven large cuspidate setae, and the upper and lower pairs largest (Figure 9A); upper margin with three pairs of simple setae and the lower margin with numerous simple setae (Figure 9B–D).

Maxilla bilobed, with a triangular distal portion with a truncated outer edge (Figure 9E), base without setae, outer edge of the distal lobe with simple setae (Figure 9F, H), the inner edges of lobes straight, and thick serrulate setae along the inner edges of lobes (Figure 9G, H).

**Figure 10.**
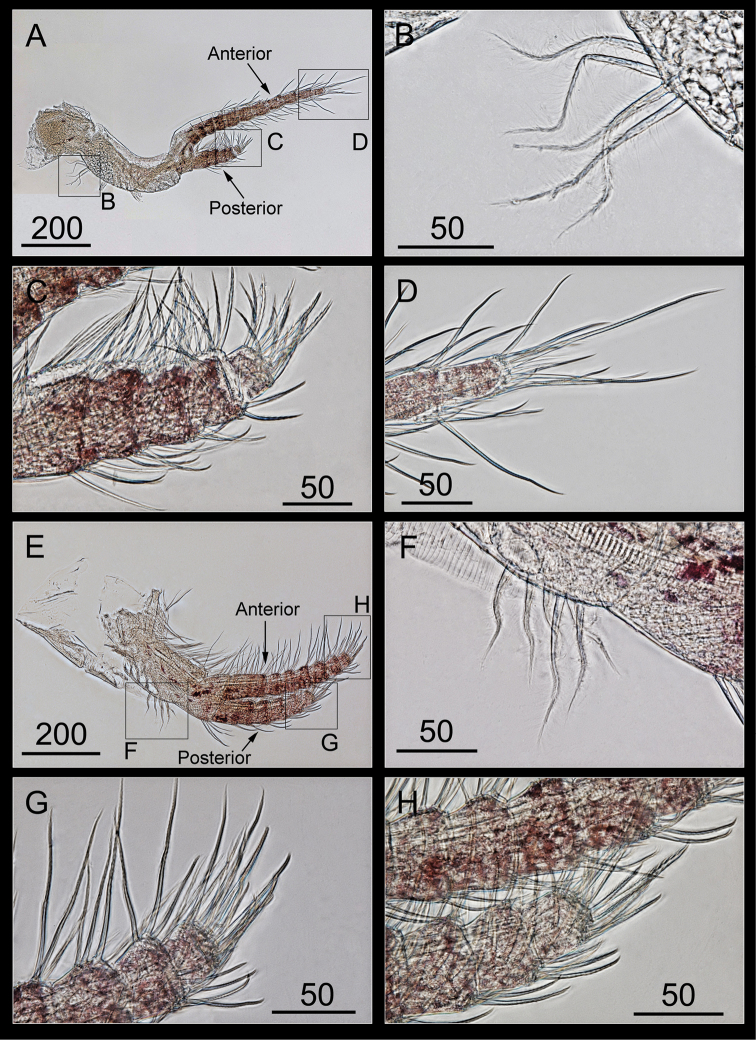
*Bryozobia rossi* sp. n. ASIZCR-000338. Cirri I (left, view from the posterior side) (**A–D**), II (left, view from the anterior side) (**E–H**). **A** cirrus I **B** tuft of setae on at base of protopod **C, D** distal segments of posterior and anterior rami **E** cirrus II **F** setae on posterior margin of protopod **G, H** distal segments of anterior and posterior rami. Scale bars in μm.

Cirrus I with unequal rami, anterior ramus with eleven segments, twice as long as the posterior ramus (five segments; Figure 10A), a protopod without setae at the anterior margin, with a tuft of plumose setae at the posterior margin (Figure 10A, B), and both the rami with serrulate setae on the intermediate segments, and bidentate and serrulate setae on the distal ends of anterior and posterior rami, respectively (Figure 10C, D).

Cirrus II with unequal rami, posterior ramus (six segments) shorter than the anterior (eight segments; Figure 10E), a protopod with plumose setae at the anterior margin and a tuft of plumose setae at the posterior margin (Figure 10F), the intermediate segments of both the rami with serrulate setae, and the distal ends of both the rami with bidentate setae (Figure 10G, H).

**Figure 11.**
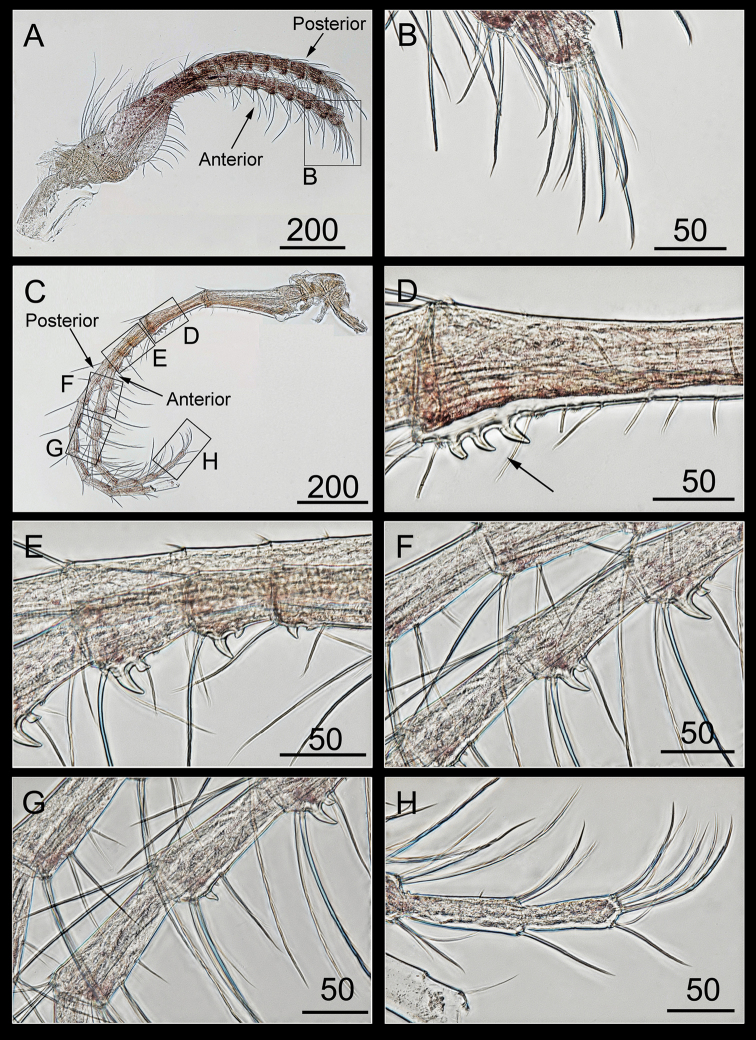
*Bryozobia rossi* sp. n. ASIZCR-000338. Cirri III (left, view from the posterior side) (**A, B**), IV (left, view from the anterior side) (**C–H**). **A** cirrus III **B** distal segment of anterior ramus **C** Cirrus IV **D** setae and denticles on basis **E, F** teeth on proximal segments of anterior ramus **G** intermediate segments of anterior and posterior rami **H** distal segments of anterior ramus. Scale bars in μm.

Cirrus III with subequal rami, a ten-segmented posterior ramus, nine-segmented anterior ramus (Figure 11A), a protopod with serrulate setae at the anterior margin and plumose setae at the posterior margin, the intermediate segments of both the rami with serrulate setae, distal ends of both the rami with bidentate and serrulate setae (Figure 11A, B).

Cirrus IV with unequal rami, a twelve-segmented anterior ramus, a posterior ramus broken with eleven segments on its remaining part (Figure 11C), a protopod with short setae having three curved teeth on the anterior margin (Figure 11D), proximal segments of the anterior ramus with one or two curved teeth (Figure 11E, F), intermediate segments of the anterior ramus with two pairs of long and short serrulate setae, intermediate segments of the posterior ramus with three pairs of long, medium, and short serrulate setae (Figure 11F, G), and the last segment of the anterior ramus with serrulate setae (Figure 11H).

**Figure 12.**
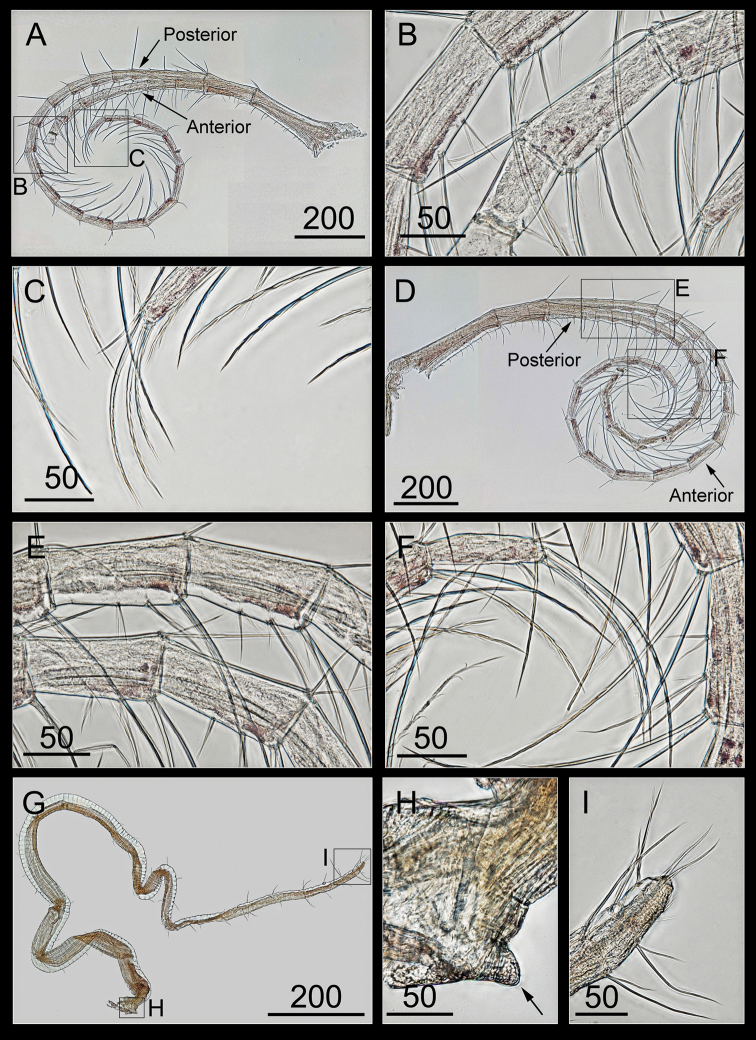
*Bryozobia rossi* sp. n. ASIZCR-000338. Cirri V (left, view from the anterior side) (**A–C**), VI (left, view from the posterior side) (**D–F**) and penis (**G–I**). **A** cirrus V **B** setae on intermediate segments of posterior and anterior rami **C** distal segment of posterior ramus **D** cirrus VI **E** setae on intermediate segments of posterior and anterior rami **F** distal segment of anterior ramus **G** penis **H** rudimentary basidorsal point **I** setae on tip. Scale bars in μm.

The cirri V and VI were similar in length, with the anterior rami of cirri V and VI both having twenty-one segments, and the posterior rami of cirri V and VI were both broken, with nine and fifteen segments on their remaining parts, respectively. A short and simple protopod was observed on the anterior margin and long serrulate setae on the posterior margin (Figure 12A, D), intermediate segments of both the rami with three pairs of long, medium, and short serrulate setae, and the last segments of both the rami with serrate setae (Figure 12B, E, C).

The penis was approximately the same length as the cirrus VI, finely annulated, gradually tapering at the tip (Figure 12G), with a vestigial basidorsal point (Figure 12H), and long scarce setae scattered along the penis (Figure 12G, I).

##### Etymology.

We named the organisms after the famous cirripedologist late Prof. Arnold Ross (Scripps Institution of Oceanography, USA), who discovered the subfamily Bryozobiinae.

##### Remarks.

All previously described specimens of *Bryozobia synaptos* from Madagascar and Mauritius and *Bryozobia* sp. from Sri Lanka possess several (two to three) more or less regular whorls of shell portals (Ross and Newman 1996, Van Syoc and Newman 2010). The interparietal portal below CL^2^ belonging to the first elevated whorl eliminates this plate; therefore, the shell becomes four plated below this whorl. All studied specimens of *Bryozobia rossi* do not have the first elevated whorl of several portals; however, only a pair of interparietal portals below CL^2^ eliminated these plates. *Bryozobia rossi* is characterized by a single irregular basal whorl of portals situated at the same level as the basal hemiportals and this differentiates the new species from the previously discovered forms of *Bryozobia*. Each whorl of shell portals is added ontogenetically and the sizes of studied specimens are similar to those studied from Indian Ocean, revealing that the new species has less number of whorls of portals compared with the previously described species. Although *Bryozobia rossi* has less number of portals and whorls of portals, it belongs to the genus *Bryozobia* because it has short, eliminated CL^2^ and open portals remaining attached to with the basal atria through tubular fillets. Opercular plates of *Bryozobia rossi* were similar to those in *Bryozobia synaptos* (absent in the specimens of *Bryozobia* from Sri Lanka).

The previously described *Bryozobia* from Madagascar, the Mascarene Plateau and Sri Lanka states that the radii between the R-CL^1^ are obsolete, whilst radii between R-CL^1^ in *Bryozobia rossi* sp. n. in the present study is well developed. In addition, the original diagnosis of *Bryozobia* from Madagascar and the Mascarene Plateau did not include parietal calcipedia, in which this character is present in *Bryozobia rossi* sp. n. In the present study, we conclude it is premature to modify the diagnosis of *Bryozobia* due to whether these discrepancies are ecotypic or specific differences is unknown. We propose to include *Bryozobia rossi* as *incertae sedis* in *Bryozobia*, deferring a decision as to whether or not it is a new genus in the Bryozobiinae when further molecular phylogenetic analysis is conducted in bryozobiine species.

The previously studied specimens of *Bryozobia* were represented by subfossil materials. The present description is the first for the morphology of the oral cone, cirri, and penis in this genus. Their morphology does not differ considerably from that in other bryozobiines, and cirrus IV with recurved teeth, characteristic of most of these barnacles. This is a first discovery of *Bryozobia* in Pacific; the previous ones were from the Indian Ocean.

## Discussion

The morphological structures, such as atria, portals, pores of basis, calcipeds, and armament of cirri IV, were attached to the teratogenesis, and the adaptations of symbiosis to the sponge are the topics predominantly discussed in the bryozobiines (Van Syoc and Newman 2010). Other sponge-inhabiting barnacles of the subfamily Acastinae, living in massive sponges that completely surround them, develop a cup-shaped base and have a greater height/width ratio of the wall plates resulting from the increasing thickness of the sponge (Kolbasov 1993). However, the bryozobiines are closely associated with encrusting/burrowing sponges which spread across the substratum as a relatively thin cortex requiring adaptations differing from those in acastines (Van Syoc and Newman 2010). These barnacles, compared with acastines, retain various connections of the rather flat base with the substratum and have an approximately conical shell. Van Syoc and Newman (2010) reported correctly that the complex system of atria and hemiportals and the portals attached to them evolved as additional space for burrowing sponge host that may prevent barnacle overgrowth. Moreover, we propose that these structures may more appropriately fix the barnacle on the sponge substratum. Bryozobiines are attached to hard substrata (mollusk shell, coral etc.) through the small central portion of the base and its thin calcipeds between the atria. In addition, the burrowing sponge growing through the atria and its fillets sealing off at the portals and hemiportals may fix a barnacle in place within the sponge host. The genus *Eoatria* having only six unperforated atria ending with six basal hemiportals develops numerous basal calcipeds of parietes for more appropriately fixing on the substratum, whereas other bryozobiines that have a developed network of perforated atria, hemiportals, and portals possess either a few calcipeds or lack them. Evidently, portals originate from hemiportals when basal parietal footings are fused. Therefore, open portals of *Bryozobia* remaining attached to the basal atria through tubular fillets are rather plesiomorphic compared with the closed portals that dissociated from the base in *Multatria*, *Microporatria*, and *Poratria* genera. Further evolution within genus *Bryozobia* was expressed in the gradually increasing number of whorls of portals from an irregular whorl in *Bryozobia rossi* to two or three regular whorls in the *Bryozobia synaptos* and *Bryozobia* sp. from Sri Lanka. The other plesiomorphic condition was the retained unfused six-plated shells observed in the *Multatria* and *Bryozobia* genera. However, *Bryozobia* having rudimentary CL^2^ appears more advanced in this character compared with *Multatria*, which possesses six plates reaching the base. The genus *Poratria* with closed portals and all fused basal shell plates may be the most evolved Bryozobiinae.

We agree with suggestion of Van Syoc and Newman (2010) that the pores of the basal atria and those of the portals of Bryozobiinae and windows (fenestrae) in Acastinae (Kolbasov 1993) may facilitate chemical interactions with the sponge to prevent overgrowth. Some acastines (*Acasta spongites* (Poli, 1791)) possess distinct and numerous pores along the growth lines of the base not organized in the radial atria; however, it may have a similar function as the pores of the base in bryozobiines. In the coral associated barnacle *Pyrgoma kuri* (family Pyrgomatidae), the base have specialized perforated furrows which these structure is believed to allow chemical mediations between the coral host and barnacle through the perforations (Roos and Newman 2000). The recurved teeth on cirri IV developed in most of bryozobiines and several acastines clean the opercular aperture off the sponge overgrowth.

Only one species of Bryozobiinae was previously reported from the studied area, namely *Eoatria quinquevittatus* (Hoek 1913) from South West Japan (Van Syoc and Newman 2010). The *Bryozobia rossi* finding in tropical and subtropical Western Pacific spreads the distribution of the genus *Bryozobia* considerably. Thus, three bryozobiine genera, namely *Eoatria*, *Multatria*, and *Bryozobia* have an Indo–West Pacific distribution, whereas *Microporatria* and *Poratria* genera are yet restricted to the equatorial zone of Western Pacific.

Van Syoc et al. (2015) revealed that *Bryozobia* is obligate with the sponges *Clathria* in the family Microcionidae. In the present study, *Bryozobia* from Taiwan was collected from the encrusting sponges *Agelas* (family Agelasidae) and *Theonella* (family Theonellidae), thus providing additional records for the family of sponges inhabited by *Bryozobia* and bryozobiines as well.

## Supplementary Material

XML Treatment for Bryozobia rossi
